# Meta-analysis discovery of tissue-specific DNA sequence motifs from mammalian gene expression data

**DOI:** 10.1186/1471-2105-7-229

**Published:** 2006-04-27

**Authors:** Bertrand R Huber, Martha L Bulyk

**Affiliations:** 1 Division of Genetics, Department of Medicine, Brigham and Women's Hospital and Harvard Medical School, Boston, MA 02115, USA; 2 Department of Pathology, Brigham and Women's Hospital and Harvard Medical School, Boston, MA 02115, USA; 3 Harvard-MIT Division of Health Sciences and Technology, Harvard Medical School, Boston, MA 02115, USA

## Abstract

**Background:**

A key step in the regulation of gene expression is the sequence-specific binding of transcription factors (TFs) to their DNA recognition sites. However, elucidating TF binding site (TFBS) motifs in higher eukaryotes has been challenging, even when employing cross-species sequence conservation. We hypothesized that for human and mouse, many orthologous genes expressed in a similarly tissue-specific manner in both human and mouse gene expression data, are likely to be co-regulated by orthologous TFs that bind to DNA sequence motifs present within noncoding sequence conserved between these genomes.

**Results:**

We performed automated motif searching and merging across four different motif finding algorithms, followed by filtering of the resulting motifs for those that contain blocks of information content. Applying this motif finding strategy to conserved noncoding regions surrounding co-expressed tissue-specific human genes allowed us to discover both previously known, and many novel candidate, regulatory DNA motifs in all 18 tissue-specific expression clusters that we examined. For previously known TFBS motifs, we observed that if a TF was expressed in the specified tissue of interest, then in most cases we identified a motif that matched its TRANSFAC motif; conversely, of all those discovered motifs that matched TRANSFAC motifs, most of the corresponding TF transcripts were expressed in the tissue(s) corresponding to the expression cluster for which the motif was found.

**Conclusion:**

Our results indicate that the integration of the results from multiple motif finding tools identifies and ranks highly more known and novel motifs than does the use of just one of these tools. In addition, we believe that our simultaneous enrichment strategies helped to identify likely human *cis *regulatory elements. A number of the discovered motifs may correspond to novel binding site motifs for as yet uncharacterized tissue-specific TFs. We expect this strategy to be useful for identifying motifs in other metazoan genomes.

## Background

A key step in the regulation of gene expression is the sequence-specific binding of TFs to their DNA recognition sites. Since transcription factor binding sites (TFBSs) are usually short (~5–15 basepairs (bp)) and a typical sequence-specific TF binds to sites that are similar to each other, a number of computational approaches have been developed to attempt to identify these sequences *in silico *[1]. We shall refer to a given sequence that a TF binds to as a 'word', and a collection of words that a given TF binds as a 'motif'. Various computational motif finders have been developed to search among an input set of sequences in order to identify over-represented DNA motifs and have been based upon a range of algorithmic approaches, such as Gibbs sampling [2], expectation maximization [3,4], and word enumeration [5-9].

At its core, motif finding is essentially a signal-to-noise problem. It has been estimated that in humans about 3% of intergenic regions are regulatory elements, whereas about 15% of intergenic regions are regulatory elements in the yeast *Saccharomyces cerevisiae *[10]. Motif finding in metazoans has also been significantly more challenging than in prokaryotes or yeast because TFBSs in metazoan genomes can be found far away from the promoter regions [1], and because the noncoding regions are typically extremely lengthy.

Additional filters have been applied to mammalian input sequences in attempts to further increase the over-representation of particular motifs within a given input set of sequences. Examination of physically enriched genomic regions, such as from microarray-based readout of chromatin immunoprecipitation (ChIP-chip, or *in vivo *genome-wide location analysis) allows the identification of regions of a genome that are bound *in vivo *by a particular TF, and thus also allows the identification of TFBS motifs [11,12], although these experiments have several limitations [13]. Of note, phylogenetic footprinting has been successful in enriching for TFBS motif matches in mammalian noncoding regions known to contain TFBSs, surrounding similarly expressed genes [1,14,15].

In addition to searching for motif matches within conserved sequence, one can perform a *de novo *motif search within conserved sequence [16] or for conserved motifs [17,18]. For example, Xie *et al. *examined promoter regions and 3' UTRs in the human, mouse, rat, and dog genomes for over-represented, conserved motifs [18]. For known regulatory motifs, they filtered TRANSFAC [19] motifs to identify those with a high motif conservation score, over the four mammalian genomes examined, relative to comparable random motifs. In order to identify novel motifs, they exhaustively enumerated all motifs 6–18 bp in length, and filtered for those with high motif conservation scores. A key issue in considering this approach is that the use of the motif conservation score relies upon the assumption that the position of a motif occurrence has been highly conserved in mammalian genomes. However, TFBSs may shuffle through evolution, such that their positions in aligned sequence are not conserved, despite the motif occurrence remaining functional [20]. Indeed, it is unclear how one should score for motif conservation to account for this phenomenon.

Here, we hypothesized that many orthologous genes expressed in a similarly tissue-specific manner in human and mouse, are likely to be co-regulated by orthologous TFs through similar *cis *regulatory regions. Therefore, we chose to perform *de novo *motif searches within human promoter regions that are conserved with the mouse genome and that are upstream of genes whose mouse orthologs exhibit tissue-specific gene expression that is highly correlated to that of their human counterparts.

**Figure 1:**
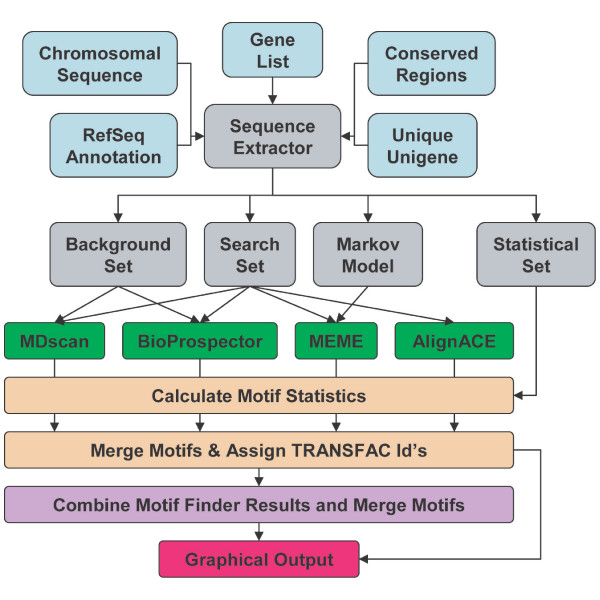
Schematic of MultiFinder design. The sequence and support files are input files (aqua boxes) required by the sequence extraction script SequenceExtractor.pl to generate a number of input files (gray boxes) required by the motif finders gray MDscan, BioProspector, MEME, and AlignACE (green boxes). Statistics are generated for the motifs, similar motifs are merged and TRANSFAC IDs are assigned (tan boxes). Motifs from the previous step are combined and similar motifs are merged (purple box). Graphical output of the combined results (purple box) from all four motif finders is generated for each scoring function (red box).

In order to automate the motif searches, we developed a software package, termed MultiFinder (shown schematically in Figure 1), that performs automated motif searching using four different profile-based motif finders, including AlignACE [21,22], MDscan [23], BioProspector [24] and MEME [4]. We anticipated that using all four of these motif finders might allow the user to combine the strengths of their different algorithms. This approach has been suggested in the context of methods for aligning noncoding sequences [25], as well as in the context of gene-finding programs [26]. Since the scoring functions from these motif finders are not directly comparable, MultiFinder allows the user to select any combination of the four motif finders and any combination of five scoring functions. Another motif analysis suite, Toucan [27], utilizes MotifSampler, a Gibbs sampling strategy, to search for motifs within conserved noncoding sequence [27], whereas MultiFinder uses a combination of motif finding programs. Another recently developed integrated system, termed RgS-Miner [28], selects motifs by the over-representation of motif pairs [28], whereas MultiFinder scores individual discovered motifs by a user-specified scoring function.

As input for MultiFinder, we used conserved regions from the alignment of the human and mouse genomes as available from the UCSC Bioinformatics website [29,30]. After motif searching, the results from each motif finder are merged and the motifs are ranked according to the user-specified scoring function. In an attempt to further enrich for mammalian regulatory motifs, we also applied a filter aimed at eliminating motifs that might score well by the various common motif-scoring metrics, but whose information content distribution does not resemble that of typical TFBS motifs.

We have applied MultiFinder successfully to yeast ChIP-chip data [11] and to a set of mammalian genes expressed in skeletal muscle [15]. MultiFinder has also been applied to conserved sequence from the alignment of *Drosophila melanogaster *and *Drosophila pseudoobscura *(data not shown). Applying MultiFinder to conserved noncoding regions surrounding co-expressed tissue-specific human and mouse genes [31,32] allowed us to discover both previously known and many novel candidate regulatory DNA motifs in all 18 tissue-specific expression clusters that we examined.

## Results and Discussion

### Motif finding within *Saccharomyces cerevisiae *sequences

We used yeast ChIP-chip data [11] as our validation data sets for evaluating MultiFinder. Specifically, we selected 10 data sets that span a range of enrichment scores in the ChIP-chip data [11] and that cover 5 gapped and 5 ungapped TFBS motifs. In order to determine the statistical significance of the motifs found by MultiFinder, 10 size-matched randomly selected sequence sets (which we shall refer to as "matched randoms") were generated for each of the 10 TFs and submitted to MultiFinder (see **Methods**). In 8 out of 10 cases (Reb1, Swi4, Yap1, Gcn4, Abf1, Rap1, Mcm1 and HSF1) MultiFinder identified the correct binding site motif, with all four motif finders identifying the correct motif as the motif with the best group specificity score [21] [see Additional Figure 1]. Despite the different motif finders' different approaches for internal representation of gaps (see **Methods**), there was essentially no difference between the motifs found by the four motif finders, although AlignACE tended to generate motifs with much better group specificity scores than did the other motif finders. Both of the TFs for which the correct motif was not identified (Pho4 and Gal4) were from sets with low enrichment scores [11]. In addition, the Gal4 motif is a particularly challenging motif for motif finders because of the 11 bp gap separating its 2 relatively short blocks of contiguous nucleotide preference. We note that it is unlikely that other types of sequence data sets, such as those from gene expression clusters, will be as highly enriched for any given TFBS motif. Determining the performance differences between the four motif finders would require a data set of intermediate difficulty; however, we chose not to focus on performing a thorough comparison of these and other possible motif finders [33], but rather on integrating the four commonly used and readily available motif finders, along with additional enrichment filters (discussed below), for use in identifying candidate mammalian regulatory motifs.

### Compensating for background word frequency variation within various classes of human noncoding regions

**Figure 2:**
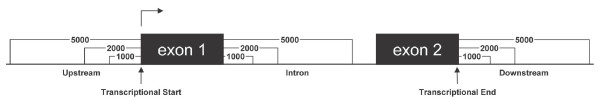
Types of genomic sequence windows examined for mammalian sequences. Three different lengths of noncoding sequence upstream, within the first introns, and downstream of genes were used to examine hexameric word frequencies.

CpG islands, which are associated with increased expression of the downstream gene, are found in the promoters of approximately 50% of mammalian genes [34]. As an illustration of the significance of this effect, we quantified the over- or under-representation of specific hexamers in each of 9 different classes of genomic regions: 0–1000 bp, 0–2000 bp and 0–5000 bp sequence windows in the immediately upstream, downstream, and intronic regions of genes (see Figure 2). Although the rankings of the word frequencies are similar among these windows [see Additional Table 1a], the relative over-representation ratios are different; this becomes more apparent when non-overlapping sequence windows are considered [see Additional Table 1b]. (We note that, interestingly, we found that the sequence windows from the first introns were enriched for GC-rich hexamers as compared to genome-wide noncoding sequence.) In order to account for the variable GC content of different region locations relative to transcription start, MultiFinder uses a background model generated specifically from the same genomic sequence window that was used for the motif search.

### Mammalian skeletal muscle cis regulatory modules (CRMs)

As a validation sequence set for MultiFinder analysis of mammalian sequence, we examined the skeletal muscle regulatory TFs Myf, Mef2, SRF, Tef1, and Sp1, and a set of experimentally verified CRMs previously collected from the literature by Wasserman *et al. *[15]. Previous phylogenetic footprinting studies demonstrated that these TFs are enriched in the evolutionarily conserved regions upstream of orthologous human and mouse skeletal muscle genes [14]. For these five TFs, there were more position weight matrix (PWM) matches for these five TFs [14,35] within the CRM sequence that is conserved between the human and mouse genomes, than there are experimentally verified binding sites (see Table 1).

In order to determine the statistical significance of the motifs found by MultiFinder, we compared them against motifs found in five sets of matched randoms. The rankings and group specificity scores of the motifs identified by the four different motif finders (see **Methods**) are shown in Table 2. MEME was the only motif finder that identified all five of the known motifs within the skeletal muscle CRMs. Although BioProspector and MDscan identified relatively few of the known motifs, they ranked the identified known motifs highest among all discovered motifs. Notably, MDscan found 372 motifs that were merged (see **Methods**) into four motif clusters and BioProspector found 361 motifs that were all matches to the Sp1 motif, whereas AlignACE found 365 motifs that were merged into 40 distinct motifs and MEME found 388 motifs that were merged into 117 distinct motifs. Thus, BioProspector and MDscan might be further aided by incorporation of a similarity metric that would eliminate very similar motifs, so that additional nonredundant motifs might be identified within the 30 and 50 motif limits imposed by MDscan and BioProspector, respectively. We suggest that when considering which motif finder(s) to use, it is important to consider how many different motifs are expected to be present in the data set.

### Filtering motifs by blocks of information content

Certain motif finders usually will find not only motifs that resemble TFBS motifs, but also numerous sequence motifs that contain individual positions of high information content interspersed with positions of low information content [see Additional Figure 2]. However, because DNA binding proteins generally interact with a number of adjacent bases of a DNA binding site [36-38], TFBS motifs are expected to contain blocks consecutive nucleotide positions with very strong base preference. This observation has been noted previously, and has been utilized in developing a new motif finding method that has been successfully applied towards identifying DNA motifs in *Escherichia coli *and *S. cerevisiae *promoters [39]. Therefore, we applied a simple filtering scheme, termed "block filtering", which removes motifs resulting from MultiFinder that failed our criteria for having blocks of information content (see **Methods**).

In order to evaluate the utility of this filtering strategy, we applied block filtering to the motifs discovered within the mammalian skeletal muscle CRM validation data set. All five of the positive control motifs, Mef2, Myf, Sp1, SRF and Tef, when initially discovered, were retained after block filtering (see Table 2). Importantly, the rankings of many of these five motifs that were discovered by AlignACE and MEME improved after block filtering, and none were eliminated by the filter. In considering all of the discovered motifs (not just the five positive control motifs), block filtering tended to eliminate motifs identified by AlignACE and MEME, suggesting that either these programs or the input parameters that we used for them tended to identify motifs that do not resemble most known vertebrate TFBS motifs.

**Figure 3:**
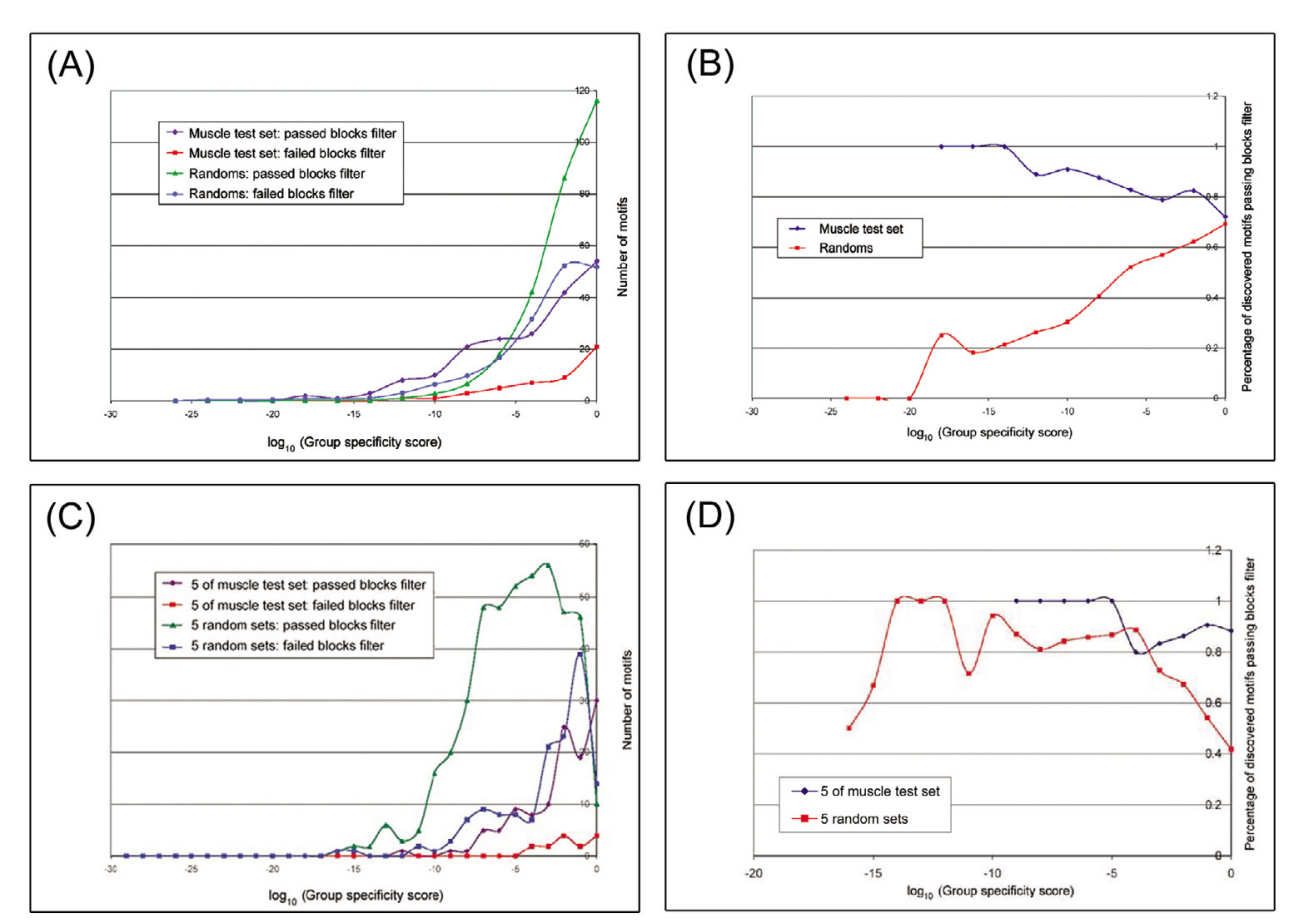
Effect of block filtering of motifs resulting from the skeletal muscle CRMs versus randomly selected sequence sets. Shown here are the nonredundant motifs from five separate MultiFinder runs for both the muscle test set and the matched randoms. **(A) **Total number of motifs, belonging to either the skeletal muscle CRM set or the size-matched set of random sequences, that either passed or failed the blocks filtering step (y-axis), at different motif group specificity cutoffs (x-axis). **(B) **Percentage of motifs, at different group specificity scores cutoffs (x-axis), that passed the block filtering step (y-axis). **(C) **As in **(A)**, except after merging motif results from 5 runs of the skeletal muscle CRM set and separately runs on 5 separate size-matched sets of random sequences. **(D) **As in **(B)**, except after merging motif results from 5 runs of the skeletal muscle CRM set and separately runs on 5 separate size-matched sets of random sequences.

In general, block filtering tended to eliminate the more group specific motifs generated from the matched randoms, and to retain the more group specific motifs found in the skeletal muscle CRMs, including known myogenic TFBS motifs (see Figure 3A,B). However, we were somewhat surprised that once we considered five separate sets of matched randoms, the number of highly group specific motifs resulting from the matched randoms far exceeded the number of comparably scoring motifs resulting from the skeletal muscle CRMs (see Figure 3C,D). This is in stark contrast to previous observations from similarly designed motif finding analyses in *S. cerevisiae*, where, for motifs exceeding a given MAP score threshold, significantly fewer motifs resulting from random sets of genes were as highly group specific as those resulting from functional categories of genes [21]. Upon inspecting the motifs from the matched randoms, we noticed that some of the highly group specific motifs appeared to be partially repetitive or long motifs of rather low information content sequences, suggesting that our block filtering criteria perhaps should be more stringent. We also noticed that some of the highly group specific motifs from the matched randoms had similarity to TRANSFAC motifs such as ETS and FOX, suggesting that non-protein-coding sequence conserved between the human and mouse genomes contains sequences that resemble core motifs for some TF families and that are frequent enough in promoter regions that the motif finders will discover them along with enough over-represented flanking sequences by chance to generate highly group specific, overfitted motifs. Overall, this analysis indicates that our comparison of group specificity scores for motifs resulting from an input tissue-specific query set versus motifs resulting from five separate sets of matched randoms is likely a conservative threshold for assigning statistical significance.

### Effect of input mammalian sequence length on motif discovery

**Figure 4:**
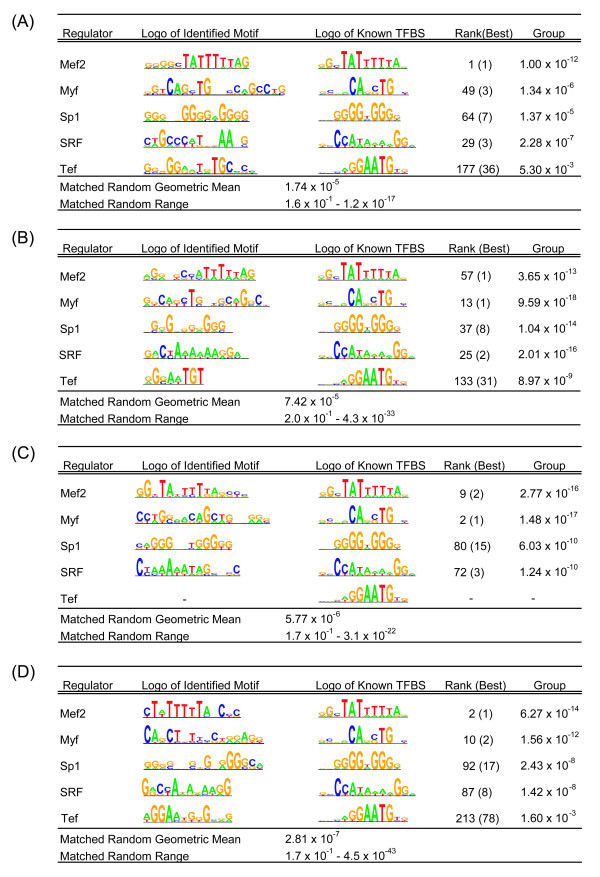
Effect of increasing surrounding sequence length on identification of the five known skeletal muscle TFBSs. TFBS motifs identified using: **(A) **the sequence conserved between the human and mouse genomes within the human CRM sequence sets previously described by Wasserman *et al. *[15]; **(B) **the sequence conserved between the human and mouse genomes within the first 1000 bp upstream of the same genes for which CRMs were analyzed in **(A)**; **(C) **as in **(B)**, except examining 2000 bp upstream of the same gene set; **(D) **as in **(B)**, except examining 5000 bp upstream of the same gene set. For each of the five TFs, the logo of the previously described motif [35] is shown, alongside the logo of the highest ranking (according to group specificity) motif matching the given TF, the discovered motif's rank and its group specificity score. Five motif finding runs were performed for each of these four input sequence sets; if a given motif was discovered multiple times over the five runs, then the ranks of both the collapsed motifs and also the highest rank from the individual runs are shown, along with the geometric mean of the group specificity scores from individual runs in which the motif was found and the logo of the best scoring motif from the individual runs. Shown below each set of discovered motifs is the geometric mean and range of the group specificity scores of motifs discovered within five size-matched sets of randomly selected regions. All results shown here are for motifs that passed our block filtering criteria.

In order to explore the effect of increasing sequence length on motif discovery (and thus presumably an increased amount of background sequence associated with the experimentally verified TFBSs (see Table 1c)), for each of the skeletal muscle genes for which we had examined CRMs (Table 2), we used as input to MultiFinder the RepeatMasked sequence conserved between the human and mouse genomes within 1 kb, 2 kb, or 5 kb upstream of their transcriptional start sites. With only a few exceptions, increasing the amount of input sequence resulted in a less significant group specificity score and thus decreased the rank of the discovered motif (see Figure 4A–D); as expected, the relative enrichment of motif matches in these regions (Table 1) was strongly correlated (r = 0.73) with motif rank. Some motifs had stronger base preference, even if not necessarily ranking higher, given a greater amount of upstream input sequence. These results also indicate that even within 5 kb upstream regions, biologically significant TFBS motifs can be found, but that these motifs will not necessarily rank particularly highly when ranked according to group specificity score. There are a number of possible reasons for this result: there may be numerous as yet uncharacterized regulatory motifs that rank more highly, group specificity may not be a suitable metric for motif discovery in mammalian genomes, indicating that transcriptional regulation requires the combinatorial action of multiple TFs, or the motif finders may still require significant algorithmic improvement to discover TFBS motifs. Since we were interested in discovering TFBS motifs from mammalian tissue-specific gene expression data, we decided to limit ourselves to the 1 kb upstream regions in subsequent motif discovery, since those regions in general resulted in TFBS motifs being ranked most highly.

### Identification of DNA motifs within mammalian tissue-specific expression clusters

**Figure 5:**
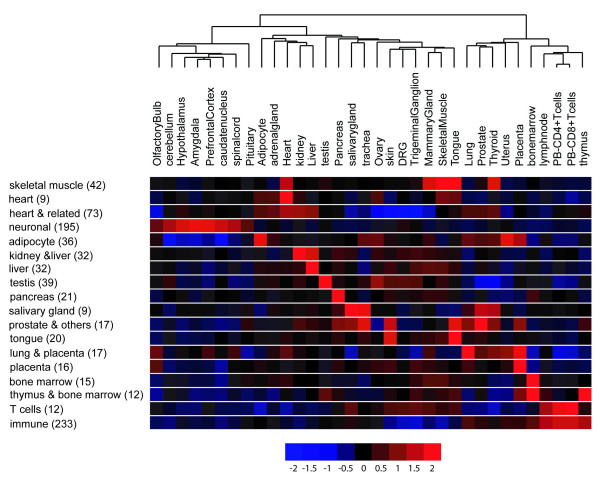
Average expression profiles for each of the 18 selected human tissue-specific gene expression clusters examined with MultiFinder. Shown along the x-axis are the 34 tissue types that were profiled for both human and mouse [32]. The color bar indicates fold-changes calculated in terms of the log_2_-transformed, normalized expression data relative to the median value over all genes and tissues.

**Figure 6:**
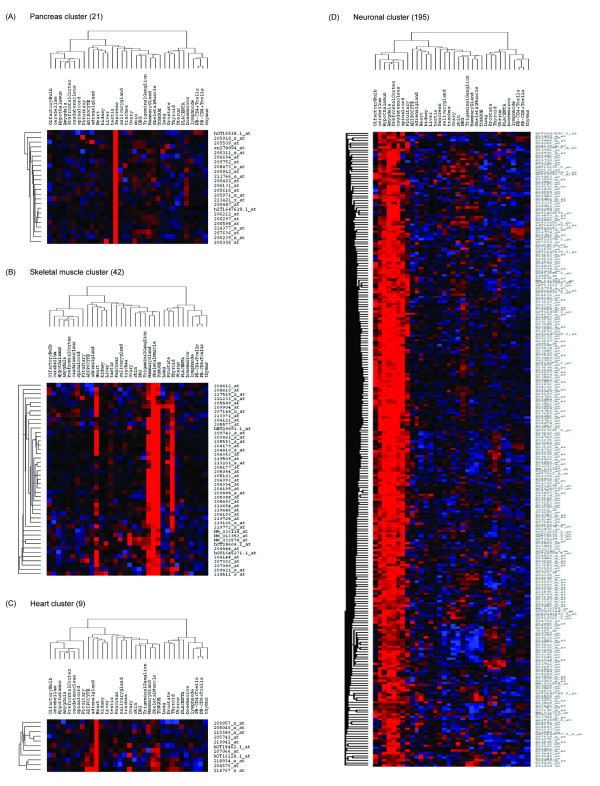
Heat maps for selected human tissue-specific gene expression clusters. **(A) **the pancreas-specific expression cluster, **(B) **the skeletal muscle-specific expression cluster, **(C) **the heart-specific expression cluster, and **(D) **the neuronal-specific expression cluster. The x-axes are as in Figure 5; shown along the y-axes are the Affymetrix accession IDs from those gene expression data sets. Note that the ordering of the tissues along the x-axis is the same for all the depicted clusters. The color bar here and in Additional Figure 1 is as in Figure 5.

The GNF SymAtlas generated by the Genomics Institute of the Novartis Research Foundation catalogs gene expression profiles from 79 human and 61 mouse tissues [32]. We clustered these data to identify groups of human genes with correlated tissue expression profiles and whose mouse orthologs also displayed similar expression patterns (see **Methods**). For motif finding, we limited ourselves to those gene expression clusters that were of biological interest to us, and for which up-regulation appeared to be quite specific for either one or a relatively small set of related tissues. The average expression profiles for each of the 18 selected human tissue-specific gene expression clusters that we examined with MultiFinder are shown in Figure 5. Heat maps for a subset of the 18 selected gene expression clusters that we refer to below are shown in Figure 6; all 18 selected gene expression clusters are shown in Additional Figure 3.

**Figure 7:**
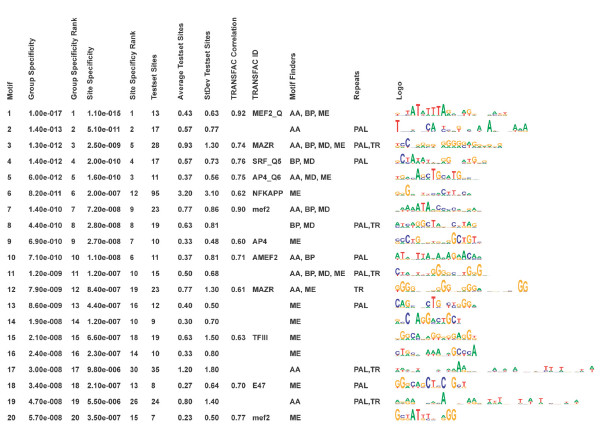
Sample MultiFinder output for the skeletal muscle gene expression cluster. Shown are just the top twenty most group specific motifs from the combined results from the four individual motif finders; many more motifs were discovered than are shown. The columns shown are as follows: group specificity score along with the motif ranking according to group specificity; site specificity score and the motif rank according to site specificity; number of motif occurrences found in the input sequence set; mean and standard deviation of the number of motif occurrences in each sequence within the input set; correlation coefficient for the most similar TRANSFAC motif and the name of the TRANSFAC motif; the motif finders (AlignACE (AA), BioProspector (BP), MDscan (MD) and MEME (ME)) that found the motif; whether a given motif contained a tandem repeat (TR) or palindromic repeat (PAL); and the sequence logo of the discovered motif.

For each of the genes in a given cluster, we searched for motifs in the conserved noncoding sequence within 1 kb upstream of transcriptional start. Since we were interested in identifying tissue-specific motifs, we assessed the statistical significance of the motifs found by MultiFinder analysis of the gene expression clusters by comparing a given motif's group specificity score [21] versus the geometric mean of the scores of all the motifs passing the block filter that resulted from analysis of five sets of matched randoms. A sample output file from a MultiFinder search of the skeletal muscle expression cluster is shown in Figure 7.

In examining the MultiFinder output, we first identified all those discovered motifs that were matches to known TFBS motifs listed in the TRANSFAC [19] Professional database (see **Methods**). Next, we explored whether any discovered motifs that are matches to TRANSFAC motifs correspond to TFs that are expressed in the given expression cluster. Here we called a TF "expressed" if its transcript's average difference (AD) value in the specified tissue was at least 200 [31] in any one of the tissues that defined the tissue specificity of the given expression cluster. For example, in examining the heart-specific expression cluster, we determined whether each TRANSFAC motif's TF was expressed in the heart mRNA samples; similarly, even if a given TF was expressed only in the cerebellum, we nevertheless called it "expressed" in the "neuronal" expression cluster. Note that it is not necessary for a TF's transcript to be present in the same RNA sample as the genes whose expression that TF regulates. For example, the TF may be expressed at very low transcript levels, or the protein may have a significantly longer half-life than does its mRNA transcript. Furthermore, it is not necessary that if a TF's transcript is present, that the active TF is actually present, with any necessary post-translational modifications, and nuclear, at the same time as its RNA is expressed. Nevertheless, we were curious to see how frequently we would actually observe that a TF is expressed in the same RNA samples as the genes that it may be regulating through the identified TFBS motif match within 1 kb of those genes' upstream sequence. In this study we focused on activators and thus gene expression clusters that exhibited tissue-specific up-regulation. A similar study could be performed focusing on repressors and clusters of down-regulated genes.

**Figure 8:**
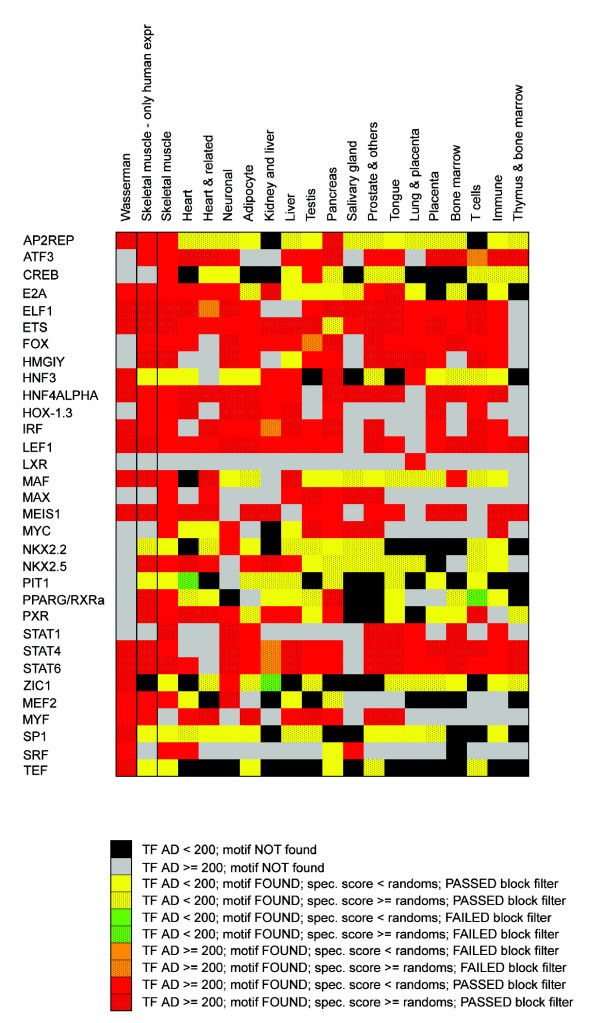
Known TFs' binding site motifs identified in tissue-specific gene expression clusters. Gene clusters are arranged in columns named according to the tissue type where the majority of the genes are up-regulated. The column labeled "Wasserman" corresponds to the 40 validated human skeletal muscle CRMs [15]; the column labeled "Skeletal muscle – only human expr" corresponds to a skeletal muscle expression cluster identified from the GNF data without considering the expression patterns of any homologous mouse genes (however, RepeatMasked, noncoding sequence conserved between human and mouse was still examined by MultiFinder). Each row represents a known TFBS motif obtained from the TRANSFAC Professional 7.4 database [69,70]. A listing of the TRANSFAC TFBS matrix accession numbers for each of the TFBS motif names shown here and all others that we considered is provided in Additional Data File 2. The Mef2, Myf, Sp1, SRF, and Tef motifs were taken from Philippakis *et al. *[35]. Shown for each expression cluster are the nonredundant motifs from five separate MultiFinder runs for both the input sequence set and the matched randoms; a correlation coefficient cutoff of 0.6 was used in the merging of highly similar motifs discovered by MultiFinder (see **Methods**). The following color scheme indicates whether a gene encoding a TF is expressed above the detection threshold (here, AD >= 200) and whether a motif matching that TF's BS motif was found by MultiFinder: black and gray boxes denote TFs whose binding site motifs we did not find, with black boxes denoting TFs that were not expressed above the detection threshold in the tissue cluster (AD < 200) and gray boxes denote those that were expressed an AD value of at least 200; yellow boxes denote TFs that were expressed below the detection threshold, but for which matches to the corresponding DNA binding site motifs were found by MultiFinder and passed the block filter; green boxes denote TFs that were expressed below the detection threshold, but for which matches to their binding site motifs were found by MultiFinder and failed the block filter; orange boxes denote TFs that were expressed above the detection threshold and were found by MultiFinder, but that failed the block filtering screen; red boxes denote TFs that were expressed above the detection threshold, were found by MultiFinder, and passed the block filtering screen; for the yellow, green, orange, and red boxes, solid colored boxes denote the discovered motifs whose group specificity scores were lower (i.e., more significant) than the geometric mean of the block-filtered motifs resulting from the size-matched randomly selected sets of genes, while the stippled boxes denote the discovered motifs whose group specificity scores were equal to or greater (i.e., less significant) than the geometric mean resulting from the size-matched randomly selected sets of genes. For the skeletal muscle CRMs ("Wasserman"), the size-matched randoms were chosen such that they were also matched to come from the same genomic regions upstream of transcriptional Start as were the Wasserman CRMs; in other words, the randoms for the 1 kb upstream Wassermansequences are all within 1 kb of the transcriptional start site. Similarly, since the examined Wasserman sequences were conserved and RepeatMasked, so too were the corresponding size-matched randoms.

**Figure 9:**
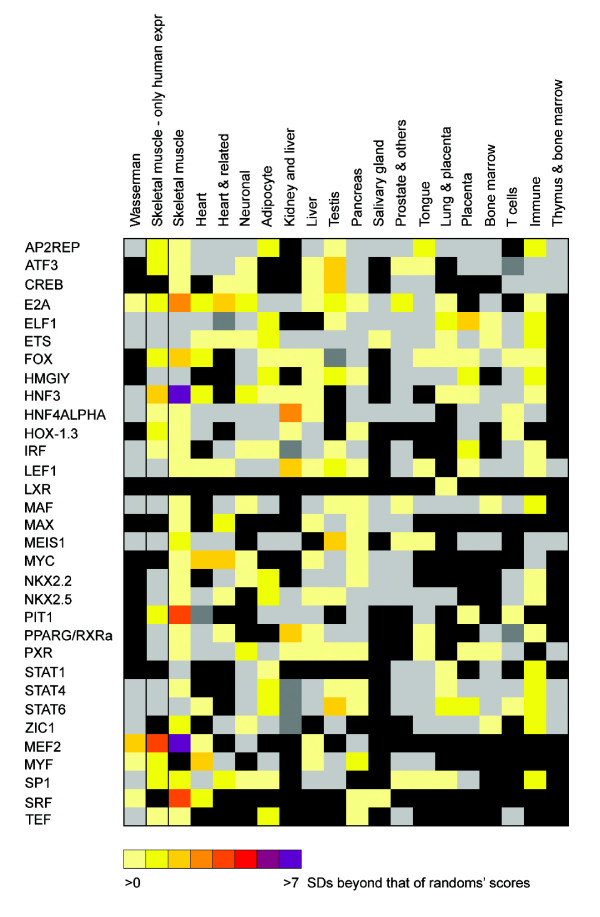
Statistical significance of known TFs' binding site motifs identified in tissue-specific gene expression clusters. Clusters are depicted as in Figure 8. Shown here is a gradient color scheme, for those motifs that both were found and also passed our block filtering criterion, indicating the significance of the motif matches in terms of standard deviations (SDs) of the log of the group specificity score for that motif match for the given input set of genes, as compared to the mean of the log of the group specificity scores for the size-matched randoms, as indicated in the color bar. Black boxes denote motif matches that were not found, dark gray boxes denote motif matches that were found but that failed block filtering, and light gray boxes denote motif matches that were found and passed block filtering, but whose group specificity scores were not more significant than the geometric mean of the size-matched randoms.

The threshold that we imposed for considering a discovered motif for potential biological significance was a group specificity score below the geometric mean of the block-filtered motifs from the matched randoms. The results of our MultiFinder analysis of the skeletal muscle CRM set, a skeletal muscle expression cluster not filtered according to expression of the mouse orthologs, and each of the 18 expression clusters are shown in Figures 8 and 9. On average, a given motif cluster mapped to 15 TRANSFAC motifs. There are at least three reasons for such a higher number of multiple mappings. First, even though we made attempts to extract only nonredundant TRANSFAC motifs based upon TRANSFAC's motif naming scheme, TRANSFAC nevertheless contains numerous very similar motifs, in some cases for the same TF, and elimination of all such redundancies would have required manual curation of their entire motif database. Second, TRANSFAC contains a number of motifs for highly homologous TFs, which are expected to have highly similar DNA binding site motifs. Third, our similarity threshold for clustering together similar motifs may be too loose and thus may be clustering together motifs that in reality should remain separate, distinct motifs.

Why does the discovery of motifs matching known TRANSFAC motifs appear to have been most successful for the skeletal muscle expression cluster, when considering the statistical significance of the group specificity scores? This result does not appear to be an effect of the size of the expression cluster, since a number of other clusters contained either many fewer or many more genes (see Figure 5). Similarly, this result does not appear to be an effect of the tissue specificity of the cluster's expression pattern, since other similarly sized clusters were of similar or even more highly tissue-specific expression [see Additional Figure 3]. Furthermore, the average pairwise correlation coefficient of the genes making up the skeletal muscle expression cluster was not higher than that for other clusters which did not exhibit highly group specific motifs (data not shown). We offer two of many potential explanations for this seemingly baffling finding: (1) the skeletal muscle tissue may have corresponded to a more homogeneous tissue type than the other tissues that were profiled; (2) the TRANSFAC motifs that we considered may be biased with more of the tissue-specific motifs being important in skeletal muscle expression.

In general, if a TF was expressed in the specified tissue of interest, then in most cases (64.0%) we identified a motif that matched its TRANSFAC motif, and many (97.5%) of those motifs discovered by MultiFinder within the given expression cluster, passed our blocks filter. It is important to remember that the presence or absence of a TF's transcript, as determined by signal intensity on the expression arrays, does not imply that the given TF was regulating its target genes in the profiled tissue samples. For example, an individual TF may be expressed but regulate its target genes only in the context of co-regulatory factors [40]. Also, even though a TF is expressed, it may be neither nuclear nor have a particular post-translational modification required for its transcriptional activity.

Conversely, of all those discovered motifs that matched TRANSFAC motifs, most (64.7%) of the corresponding TF transcripts were expressed in the tissue(s) corresponding to the expression cluster for which the motif was found. There are a number of possible reasons why more of the corresponding transcripts were not identified as expressed. First, many TFs are known to be expressed at relatively low transcript levels and may not have AD values >200. Second, a TF may not be regulated at the transcriptional level, but rather may have a long half-life and in addition may be regulated post-translationally. Third, an important caveat in interpreting these results is that there may be more than one TF that is capable of binding the discovered sequence motif. Indeed, a number of the TRANSFAC motifs are not TFBS motifs for one specific TF, but rather for a class of TFs, such as ETS or FOX; for example, the FOX matrix, is composed of binding sites for 15 different FOX family members. Moreover, the differences between a motif discovered by MultiFinder and the TRANSFAC motif for the same TF may be significant enough to prevent identification. Here, we used a reduced set of motifs generated from TRANSFAC Professional version 7.4 (see **Methods**). Interestingly, of the discovered motifs that matched TRANSFAC motifs and whose corresponding TFs were expressed, many (49.2%) had a group specificity score more significant (i.e., lower) than that for the size-matched randoms, with some motifs scoring multiple standard deviations better. This suggests that many of the motifs discovered by MultiFinder are not only statistically significant, but may also be biologically important. Finally, for about one-third (36.0%) of those TFs that were expressed, we did not discover their binding site motifs within that given expression cluster. These might correspond to false negatives of MultiFinder. Alternatively, these motifs might be found beyond the 1 kb upstream regions that we examined in this study, or these TFs might not contribute towards the tissue-specific gene expression patterns of these clusters, despite their being expressed in those clusters. We note that almost all of those motifs for which the corresponding TF was expressed, passed our block filter (97.5%).

This analysis of motifs matching known TRANSFAC motifs resulted in a number of interesting findings, which suggest that our overall strategy for discovering sequence motifs in mammalian gene expression data is promising. For example, the homeodomain TF NKX2.2 is expressed in the ventral central nervous system and is known to regulate the differentiation of oligodendrocytes in spinal cord [41]. Within the pancreas, NKX2.2 is required for the differentiation of pancreatic beta cells, and thus has been implicated in diabetes [42]. Only in the neuronal gene expression cluster was NKX2.2 expressed, and was a motif discovered that matched the NKX2.2 TRANSFAC motif. A motif matching the NKX2.2 motif was also discovered in pancreas, although in pancreas the NKX2.2 transcript was present at an AD value below 200.

It is important to note that motif matches do not necessarily indicate direct regulation by the indicated TF, but rather simply indicate sequence matches beyond the similarity threshold. For example, the NKX2.2 motif was also discovered in the skeletal muscle, adipocyte, and immune gene expression clusters, although in these tissues NKX2.2 was present at an AD value below 200. Given that muscle and adipose tissue have previously been shown to be involved in glucose homeostasis [43,44], we were curious whether NKX2.2 might have an as yet undescribed role in these tissues in regulating genes involved in glucose homeostasis. However, these expression clusters did not have an over-representation of Gene Ontology [45] annotation terms pertaining to glucose homeostasis. Therefore, some other TF that binds a motif similar to the NKX2.2 motif might actually regulate genes in these expression clusters. Indeed, a Pfam search indicates that there are 277 homeobox proteins in the human genome (data not shown). It is quite possible that TFs of the same structural class and with a high degree of sequence similarity in their DNA binding domains might potentially have similar DNA binding site specificities.

The TF peroxisome proliferator-activated receptor gamma (PPARgamma) provides another interesting example. PPARgamma is a nuclear hormone receptor that heterodimerizes with retinoid X receptor (RXR) alpha. PPARgamma is expressed predominantly in adipose tissue, and also in the lower intestine and in cells involved in immunity. It is of particular biomedical interest because it is thought to help maintain proper levels of key glucoregulatory and lipogenic molecules, and has been implicated both in diabetes and obesity [46]. Indeed, adipose-specific deletion of PPARgamma in mice causes insulin resistance in fat and liver [47]. PPARgamma has also been shown to directly activate genes in the pancreas and liver that are important for glucose-sensing [48]. Interestingly, expression of PPARgamma in skeletal muscle has been shown to be important for maintenance of insulin action in skeletal muscle in mice [49]. Dominant-negative mutations in human PPARgamma are associated with severe insulin resistance, diabetes mellitus, and hypertension [50]. Interestingly in all tissues where PPARgamma is expressed, the RXRalpha gene is also expressed (the converse is not true; RXR is expressed in most of the tissues in the GNF data set, and it is known that it serves as a heterodimerization partner for many nuclear receptors [51]).

The TRANSFAC motif for PPARgamma is actually the motif for the PPARgamma/RXRalpha heterodimer, which is a highly information-rich motif containing 12 positions of very strong nucleotide preference, and so it is very likely that a discovered motif matching this TRANSFAC motif is a true motif match. Using MultiFinder, we discovered the PPARgamma/RXRalpha binding site motif, with a group specificity score surpassing that of the size-matched randoms, in the skeletal muscle, heart & related, kidney & liver, liver, tongue, and immune gene expression clusters, consistent with the known biological roles of PPARgamma. We found it surprising that we did not discover the PPARgamma motif in the adipocyte cluster, given the importance of PPARgamma in adipose tissue [46,47]. It is possible that the genes in the adipocyte cluster were not actually highly enriched for genes that are regulated directly by PPARgamma. Alternatively, in this cluster of genes PPARgamma may bind a motif that is not a close match to the TRANSFAC PPARgamma/RXRalpha motif. We also discovered the PPARgamma/RXRalpha motif in the heart, testis, pancreas, and placenta expression clusters, although the group specificity scores were not significant for these clusters. Of note, the DNA binding site for the PPARgamma/RXRalpha heterodimer is a direct repeat, referred to as "direct repeat 1 (DR1)" [52]. Nuclear receptors generally bind to DNA as either heterodimers or homodimers; the DR1 half-site is capable of binding many other nuclear receptors, including the thyroid hormone receptor and the vitamin D receptor [51]. Thus, lack of significant group specificity scores in these tissues may be due to the occurrence of TFBSs for other similar nuclear receptors, resulting in partial matches to the DR1 motifs. Of the tissue-specific gene expression clusters in which we found the PPARgamma/RXRalpha motif, PPARgamma was expressed in skeletal muscle and pancreas, suggesting that PPARgamma exerts a more significant regulatory role in skeletal muscle and pancreas for maintaining glucose homeostasis than has been described thus far. In addition, the discovery of the PPARgamma/RXRalpha motif in the testis expression cluster may provide insight into the involvement of PPARgamma in testicular cancer [53].

**Figure 10:**
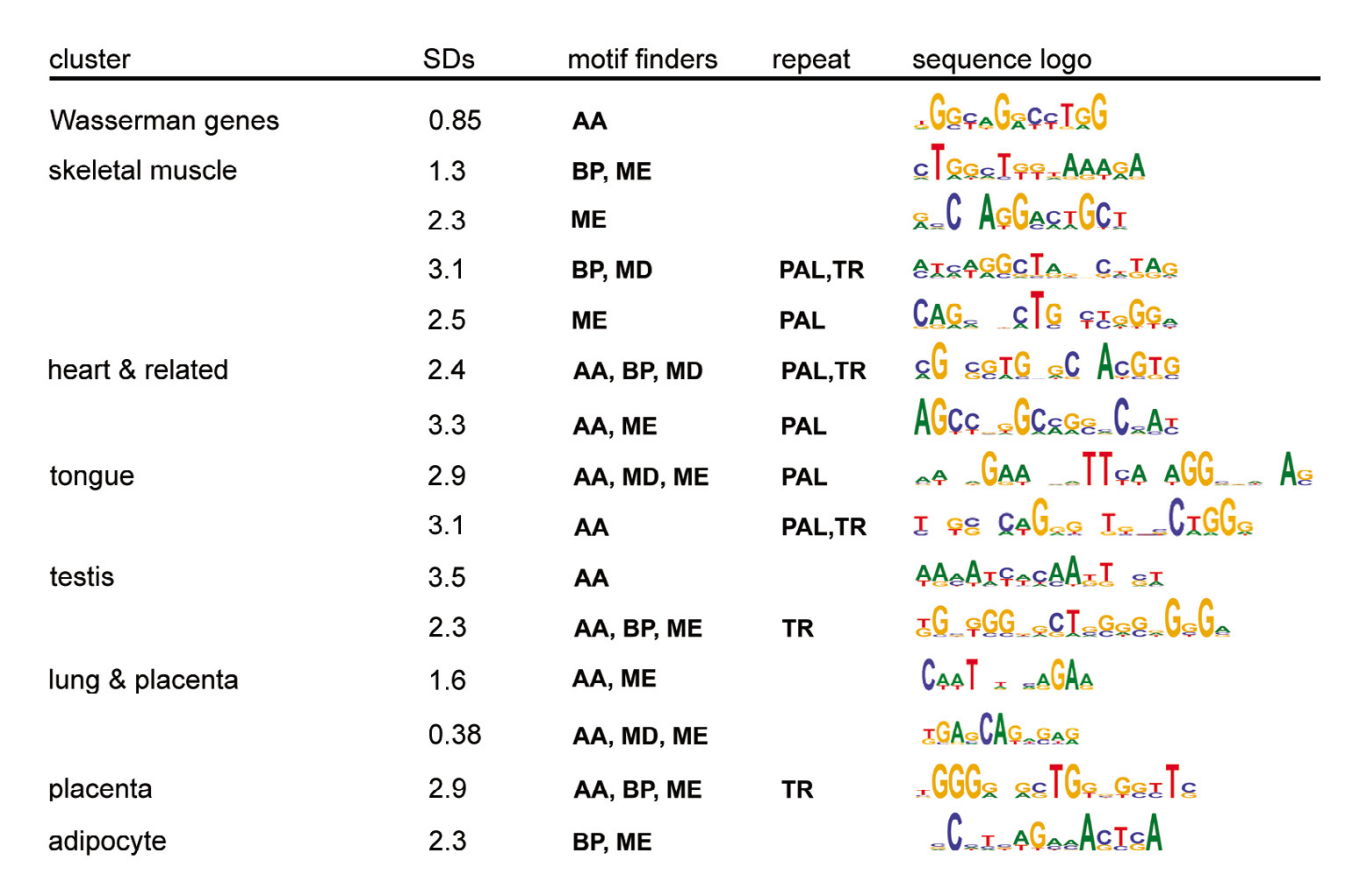
Examples of discovered, novel human tissue-specific DNA sequence motifs. Shown are the clusters that yielded the motif; the number of SDs of the log of the group specificity score for that motif match for the given input set of genes as compared to the mean of the log of the group specificity scores for the size-matched randoms, the motif finder(s) (AlignACE (AA), BioProspector (BP), MDscan (MD) and MEME (ME)) which discovered the motif; whether a given motif contained a tandem repeat (TR) or palindromic repeat (PAL); and the sequence logo of the discovered motif.

In addition to identifying previously known TFBS motifs, we also discovered a large number of novel, candidate TFBS motifs. In total, over the 18 expression clusters that we examined, we discovered 431 previously known TFBS motifs and 579 novel, nonredundant motifs with group specificity scores better than the geometric mean of their corresponding matched randoms. Examples of some newly discovered, candidate regulatory motifs are shown in Figure 10; complete MultiFinder results for all the known and novel motifs are available online at our website  and in Additional Data File 3. Interestingly, a number of the novel motifs contained within them either a tandem repeat or palindromic sequence, or in some cases, both of these. We may be able to evaluate the functions of some of these motifs in future analyses, such as by assessing their co-occurrence with known regulatory motifs [35,54]. High-throughput genomic technologies such as one-hybrid assays [55] may help to establish what TF(s) bind to these candidate regulatory motifs.

Although Xie *et al. *discovered tissue-specific motifs, their search strategy was to perform a search for over-represented *k*-mers over all ~2 kb promoters, and then to map those *k*-mers back to tissue-specific expression data. They discovered 69 previously known TFBS motifs, and 105 novel motifs. In contrast, our goal was to use tissue-specific expression data to discover motifs, and thus also to demonstrate that an expression dataset focused on one or a small number of related tissues could be used to identify candidate regulatory motifs for those tissues. Nevertheless, on average 26% of the tissue-specific motifs that we discovered within a given expression cluster were also found by Xie *et al.*, although this fraction drops to 3.2% if we also require that the Xie *et al. *motifs were found by those authors to be enriched within at least one of the same tissue(s) that defined the tissue-specificity of our expression clusters. Similarly, of the motifs found by Xie *et al. *to be enriched within tissue(s) defining one of our 18 expression clusters, on average 42% were found by MultiFinder and had a group specificity score better than the geometric mean of the matched randoms. None of the 105 novel motifs discovered by Xie et *al. *were found among our novel, block-filtered motifs that scored beyond the geometric mean of the matched randoms. Since our study was focused on identifying tissue-specific motifs within 18 expression clusters, it is quite possible that in effect we were searching for less common motifs, that would have been unlikely to have been found in Xie *et al.*'s genome-wide search for over-represented *k*-mers.

In order to assess the improvement in motif discovery afforded by our ortholog co-expression filter, we separately clustered all of the human GNF expression data, rather than just those genes whose mouse orthologs displayed similar expression patterns. From these data, we chose to examine an expression cluster that exhibited skeletal muscle-specific up-regulation [see Additional Figure 3]. Motif finding was then performed on those genes' 1 kb upstream, RepeatMasked sequences conserved between the human and mouse genomes, just as for all the other expression clusters. Results from this comparison indicate that imposing the co-expression restriction tends to result in motifs with more group specific scores than when considering human expression data alone (see Figure 9), although some motifs may be missed because a smaller input gene set is used then.

### Integrating results from a combination of motif prediction tools

The four motif finders that we used in this present study exhibited tendencies for identifying different types of motifs, in part because they impose a different penalties on opening gaps within blocks of sequence preference. In our study, AlignACE and MEME tended to find longer motifs, while MDscan and BioProspector tended to find more compact motifs. Block filtering tended to eliminate more AlignACE and BioProspector motifs. BioProspector and MDscan also tended to find fewer distinct motifs in a given data set. As shown in Table 2, within the skeletal muscle CRMs AlignACE and MEME identified more known motifs than did BioProspector and MDscan, suggesting that AlignACE and MEME may be advantageous when multiple motifs are present in an input sequence set, while BioProspector and MDscan may be better for making reliable predictions of the most frequently occurring motif(s). BioProspector and MDscan were also faster than AlignACE or MEME.

## Conclusion

In this study, we searched within sequence conserved in alignment of the human and mouse genomes. As more mammalian genome sequences become available, we will be able to further enrich for likely regulatory regions by limiting our motif searches to those regions that are conserved across all the mammalian genomes. As long as species- or lineage-specific biological processes are not being explored, we can expect this strategy in general to be helpful. In addition, as more gene expression profiling experiments are performed and deposited in public databases, a greater range of tissue types can be examined for shared expression patterns across multiple species. Moreover, it would be very useful to develop an algorithm for considering conservation of a motif occurrence, while allowing the occurrence to be rearranged in the aligned genomic sequences.

Recently Tompa and colleagues organized a motif finding competition, in which the performance of 13 different motif finders was compared using a variety of real and synthetic sequence sets covering a range of genomes [33]. A caveat for interpreting the results of this competition is that each motif finder was allowed to select only one 'best' predicted motif for each input sequence set. Nevertheless, a major conclusion from this comparison was that no single motif finder consistently outperformed the others. Moreover, the results indicate that a pairwise combination of motif finders can result in improvement over the use of a single motif finder, although the choice of motif finders is important [33]. In that study, the combination of AlignACE and MEME resulted in only marginal improvement; unfortunately MDscan and BioProspector were not included in this comparison. Our results also indicate that the integration of the results from these four motif finding tools identifies and ranks highly more known and novels than does the use of just one these tools. Although it is possible that only a subset of these four motif finders may be sufficient to gain this improvement, or that some other combination of multiple motif finders may be optimal, it was not our goal to identify such an ideal combination. In addition, we found that the incorporation of our 'block filter' tended to eliminate motifs that in some cases scored well in terms of group specificity, but whose information content distribution does not resemble that of most known TFBS motifs.

It would also be of interest to examine motifs in more than just the 1 kb of upstream sequences for particular gene expression clusters. Despite our various strategies for enriching for likely similar regulatory regions, our comparison of motif finding in 1 kb versus 2 kb versus 5 kb of upstream sequence for the a set of skeletal muscle genes indicates that there is still much improvement that remains to be made in identifying even the known TFBS motifs with high confidence. Since TFBSs are often spatially clustered into *cis *regulatory modules that regulate gene expression in a temporal and tissue-specific manner [1], analysis of both the known and novel discovered motifs using a tool such ModuleFinder [35] may allow not only the identification of candidate *cis *regulatory modules but also the assignment of even higher confidence to those novel motifs that are found to significantly co-occur with known TFBS motifs [54]. In addition to performing *de novo *motif searches on conserved noncoding sequence, one can also further restrict motif searches to known or suspected regulatory regions. For example, searching for motifs within a set of known or predicted CRMs [56] may be a particularly powerful way to increase the likelihood of discovering motifs that are important for specifying the particular gene expression pattern of interest.

## Methods

### SequenceExtractor.pl

We obtained genome sequence alignments and annotation files from the UCSC Genome Bioinformatics site [29,30]. The genomes available from this site are generally repeat masked with RepeatMasker [57] using the more stringent "-s" masking flag and thus can be used without further masking. SequenceExtractor is a Perl script that parses a subset of the annotation files from the UCSC Genome Bioinformatics site in order to extract sequence from the assembled genomes. SequenceExtractor was designed to extract the conserved human sequence from the alignments of human and mouse genome, but can also be used with other genome alignments, including multiple alignments.

As input for extracting user-defined regions of the genome, SequenceExtractor requires the user's input set of RefSeq accession numbers to be used in motif searching, the RefSeq annotation (RefFlat.txt) from UCSC, a list of nonredundant RefSeq accession numbers derived from UniGene, assembled chromosomes from UCSC and a list of all conserved regions found in each chromosome. For example, a user could choose to obtain only conserved sequence that has been RepeatMasked and is within 1000 bp upstream of the transcriptional start site. SequenceExtractor creates a graphical output file in which these various sequences regions are displayed [see Additional Figure 4 for a sample output file]. SequenceExtractor also generates a number of support files (described below) necessary for the different motif finders. A background file, used to calculate a Markov background model of an order defined by the user (usually fifth order), is generated from all of the nonredundant RefSeq accession number in UniGene. A sequence file in which non-conserved regions and masked regions are indicated by "N"s, is also generated using all nonredundant RefSeq accession numbers in Unigene; this file maintains the relative spacing of the sequence relative to the transcriptional start site, and thus can be readily used both for calculating group specificity scores and positional bias scores using the method described by Hughes *et al*. [21]. Note that MultiFinder can be run independently of SequenceExtractor and UCSC annotation.

Included as part of SequenceExtractor is an option to create a user-specified number of randomly selected input sequence sets, matched in size and position to the given input sequence set of interest (which we refer to as "matched randoms"). For example, a user inputting 20 genes whose 1 kb upstream sequence is to be searched for motifs, can specify that an additional five sets of 20 randomly selected genes also be searched for motifs within the 1 kb of upstream sequence. The use of such matched random sets has been described before [21], and is important for assessing the statistical significance of any motifs discovered within the input set of interest. For random sequence matching, SequenceExtractor first selects a random sequence of the same type as the query test set (e.g, 1000 bp upstream, RepeatMasked, conserved) that is the same size as or larger than the corresponding sequence from the query test set, and then within that region selects a randomly positioned subsequence that is exactly the same length as the sequence from the input set. This iterative process builds random sequence sets that contain the same number of sequences of the same lengths as the query test set. Attempts to generate random sequence sets containing conserved sequences that were in the exactly same position relative to transcriptional start site failed in situations where no suitable sequence existed or became exceedingly time-consuming when only a few sequences in the genome were suitable. The random sequence matcher will use a given RefSeq accession number no more than once in each matched set of randomly selected sequences. SequenceExtractor was also designed to generate randoms from a user-supplied input sequence set where the region that the sequence set came from is known but the exact positions of the sequences relative to transcriptional start site might not be known.

### Word frequency analysis

Word frequencies for the upstream, intronic and downstream regions of all nonredundant representative UniGene entries with RefSeq accession numbers were generated using the hg16 assembly of the human genome [29,30,58] and build #173 of UniGene [59]. The file containing the nonredundant representative members of each UniGene cluster (Hs.seq.uniq.Z) was scanned for RefSeq accession numbers. The sequences of interest for the RefSeq accession numbers were generated from the hg16 assembly of the human genome. Annotation listing conserved regions between the human genome (hg16) and the mouse genome (mm4), transcriptional Start/Stop sites, intron/exon boundaries and assembled chromosomal sequence for the human genome was obtained from the UCSC Genome Bioinformatics site [29,30]. The word frequency calculations were limited to the RepeatMasked human sequence that was conserved with mouse. Repeats in these sequences were masked using the "-s" setting of RepeatMasker [60]. For the upstream, intronic and downstream regions five different sequence windows were considered: 0–1000 bp, 0–2000 bp, 0–5000 bp, 1000–2000 bp and 2000–5000 bp. For comparison the word frequencies for all noncoding sequence in the human genome were generated. The ratio of the word frequency in each sequence window versus the word frequency from all noncoding sequence was calculated.

### MultiFinder.pl

MultiFinder is a Perl package that performs motif searches using up to four different profile-based motif finders, and is freely available to academic and non-profit users. The motif finders included in MultiFinder are AlignACE, MDscan, BioProspector and MEME. Our goal in incorporating motif finders into MultiFinder was to get a set of dissimilar search algorithms that would maximize the number of different motif finding algorithms used. The motif finders also had to output a motif profile that could be used to generate PWMs. Finally, the motif finders had to be readily available so that others would be able to download and use them locally. AlignACE [21,22] is a Gibbs sampling algorithm that uses GC content to model the background. BioProspector [24] is also a Gibbs sampling algorithm, but incorporates a third-order Markov model to approximate the background. MDscan [23] is a word enumeration algorithm that also uses a third-order Markov model of the background. Both MDscan and BioProspector incorporate a threshold sampler during the motif finding process that screens potential words against the evolving motif to speed up the motif finding procedure. MEME [4] is an expectation maximization algorithm and uses a Markov background model of an order chosen by the user. These motif finders all use a FASTA-formatted input file but have different support file requirements. BioProspector and MDscan both require a FASTA-formatted background file that is internally converted into a third order Markov model. MEME requires a pre-generated Markov model of background. SequenceExtractor automatically generates these sequence and support files. These files are then submitted to MultiFinder, which performs a motif search at each motif width with all of the motif finders selected by the user. The user may select a range of motif widths between 4 and 25 bp; these motif width limits are imposed by MDscan. We note that MDscan was designed to be used on ChIP-chip data or other sets of data for which each sequence has been assigned a score that can be used as a measure of how important it is likely to be in subsequent motif searches; however, we did not use this feature of MDscan in submitting the skeletal muscle CRM sequences.

These motif finders use different target scoring functions for motif finding. AlignACE, BioProspector and MDscan use alternate approximations of the maximum a posteriori (MAP) score of Liu *et al*. [61]. MEME calculates a p-value for each motif. Since the different target scoring function used by each motif finder prevent direct comparison of the scores generated by the motif finders, four other motif scoring functions were incorporated into MultiFinder, in order to allow users to pick which score(s) they wish to calculate and which one they wish to use for ranking the motifs identified from a search of the user-supplied gene list. Since our goal was to identify tissue-specific motifs, we chose to report the group specificity and site specificity scores, and to rank the motifs according to group specificity.

In this study, we chose to search a motif width range of 6–18 bp for all MultiFinder runs, allowing up to 30 motifs to be identified by each of the four motif finders. We ran each motif finder with its own default parameters. For AlignACE, the GC content for background was set based on the calculated percentage of GC from the given background file, the expect score was set to the width of the motif, the oversample was set to 5, and the minpass was set to 200. For BioProspector, the number of refinements was set to 40, the maximum gap width was set to one-third the width of the motif width, and the minimal block size was set to one-third the motif width. For MDscan, the number of motifs to refine was set to 50, the number of sequences used in the initial refinement was set to 20, and both strands were used in the motif search. For MEME, both strands of DNA were searched, the motif could occur zero times or many times in each sequence, the minimal number of words per motif was set to 10, and a fifth order Markov background was generated from the sequence background file. MEME was the only motif finder of the four that incorporated a user-defined Markov background model.

Each of these motif finders uses a different internal representation of gaps within motifs. AlignACE internally represent motifs as a collection of active and inactive positions. The inactive positions in motifs are not optimized during the Gibbs sampling phase of the motif search and tend to have little or no sequence preference. BioProspector allows an explicitly defined gap ranging between a minimum and maximum width defined by the user. During the sampling phase positions within the gap are not used to optimize the motif target function. The regions of the motif flanking the gap are used to optimize the motif. MDscan and MEME do not have a user-defined gap width parameter.

MultiFinder merges the motifs found by each motif finder across different motif widths. The merged motifs from each motif finder are then merged across all of the different motif finders. Merging motifs is done for each scoring function of interest. Motifs are merged by first using CompareACE [21] to calculate the Pearson correlation coefficients between each pair of motif PWMs, and then performing hierarchical clustering of the motifs with Tree [21]. In merging, only the best scoring member of each motif cluster is retained, thus eliminating the poorer scoring redundant motifs. After the motifs are merged, motif rank order is determined for the five motif scoring functions. The merged motif lists are output in text and graphical format for each motif finder and for each scoring function of interest, both before and after combining and merging across the different motif finders.

Motifs that were either palindromic or that contained tandem repeats were identified as blocks of sequence containing three or more contiguous nucleotide positions of at least 0.5 bits of information at each position, that were either tandem or inverted repeats of each other. Specifically, a correlation coefficient was calculated for all possible pairs of blocks, in both orientations, within each motif. Tandem repeats were defined as two blocks with a Pearson correlation coefficient of at least 0.7 with the same relative orientation. Palindromic motifs were defined by their containing two blocks with a Pearson correlation coefficient of at least 0.7, in an inverted orientation.

### BlockFilter.pl

We defined a "block" as three or more consecutive bases of at least 0.5 bits of information [37] at each position, and then identified such blocks within a motif in order to classify a motif as either ungapped or gapped. An ungapped motif was defined as a motif with a single block of four or more consecutive bases, and a gapped motif was defined as a motif containing two or more blocks that were each three or more consecutive bases long. BlockFilter.pl is a Perl script, available as part of MultiFinder, that removes motifs that do not fall into either of these classes. We applied block filtering only to the mammalian motif searches.

### Motif scoring

The four motif finders use different scoring functions in identifying motifs. These functions are not readily comparable to one another and thus cannot be used to rank the results between different motif finders. Therefore, a number of other scores that are comparable across the motif finders are automatically generated by MultiFinder. The locations of sites that match motifs are found with ScanACE [21], which searches a user-supplied sequence set for occurrences of a motif using a mononucleotide PWM representation [62] of the motif. All matches scoring better than a user-specified threshold are returned. MultiFinder calculates the group specificity score [21,63] for each motif using the hypergeometric distribution:

Sgroup=∑x=1min⁡(S,H)(Sx)(N−SH−x)(NH)
 MathType@MTEF@5@5@+=feaafiart1ev1aaatCvAUfKttLearuWrP9MDH5MBPbIqV92AaeXatLxBI9gBaebbnrfifHhDYfgasaacH8akY=wiFfYdH8Gipec8Eeeu0xXdbba9frFj0=OqFfea0dXdd9vqai=hGuQ8kuc9pgc9s8qqaq=dirpe0xb9q8qiLsFr0=vr0=vr0dc8meaabaqaciaacaGaaeqabaqabeGadaaakeaacqqGtbWudaWgaaWcbaGaee4zaCMaeeOCaiNaee4Ba8MaeeyDauNaeeiCaahabeaakiabg2da9maaqahabaWaaSaaaeaadaqadaqaauaabeqaceaaaeaacqqGtbWuaeaacqqG4baEaaaacaGLOaGaayzkaaWaaeWaaeaafaqabeGabaaabaGaeeOta4KaeyOeI0Iaee4uamfabaGaeeisaGKaeyOeI0IaeeiEaGhaaaGaayjkaiaawMcaaaqaamaabmaabaqbaeqabiqaaaqaaiabb6eaobqaaiabbIeaibaaaiaawIcacaGLPaaaaaaaleaacqqG4baEcqGH9aqpcqaIXaqmaeaacyGGTbqBcqGGPbqAcqGGUbGBdaqadaqaaiabbofatjabbYcaSiabbIeaibGaayjkaiaawMcaaaqdcqGHris5aaaa@54F7@

where *S *is the number of genes that contain words that are part of the motif, *H *is the number of target genes used by the motif finders to find the motif, *x *is the intersection between *S *and *H*, and *N *is total number of genes in the background sequence set of genes. The related site specificity score [64] is also calculated using the hypergeometric distribution. Unlike the group specificity score, the site specificity score accounts for more than one occurrence of a given motif in the input sequence windows for the input genes. This score may potentially be more useful than the group specificity score for motif finding in higher eukaryotic genomes, in which longer intergenic sequences frequently contain multiple occurrences of TFBSs. Moreover, the group specificity score becomes a poor tool for statistical comparison when most of the sequences in both the foreground and background sequence sets have an occurrence of the motif.

A ratio of the frequency of the motif in the test set versus the frequency of the motif in the background is also calculated:

O=ftestfbackground
 MathType@MTEF@5@5@+=feaafiart1ev1aaatCvAUfKttLearuWrP9MDH5MBPbIqV92AaeXatLxBI9gBaebbnrfifHhDYfgasaacH8akY=wiFfYdH8Gipec8Eeeu0xXdbba9frFj0=OqFfea0dXdd9vqai=hGuQ8kuc9pgc9s8qqaq=dirpe0xb9q8qiLsFr0=vr0=vr0dc8meaabaqaciaacaGaaeqabaqabeGadaaakeaacqWGpbWtcqGH9aqpdaWcaaqaaiabdAgaMnaaBaaaleaacqWG0baDcqWGLbqzcqWGZbWCcqWG0baDaeqaaaGcbaGaemOzay2aaSbaaSqaaiabdkgaIjabdggaHjabdogaJjabdUgaRjabdEgaNjabdkhaYjabd+gaVjabdwha1jabd6gaUjabdsgaKbqabaaaaaaa@4533@

The bit score of each motif [37] quantifies the amount of information contained in a motif using the cumulative total of information at each position of the motif:

*I*(*l*) = 2 - (*U*(*l*) + *e*(*n*))

where *U*(*l*) is the uncertainty at each position and *e*(*n*) is a correction factor that is important when there are few (*n*) sample sequences.

Bit scores tend to score long, low complexity motifs such as AT-rich regions that have not been fully masked by RepeatMasker, as being very significant. Therefore, the bit score may be less useful for assessing the regulatory relevance of a motif.

Finally, a user can also opt to report the maximum a posteriori (MAP) score, as calculated by AlignACE [21,22], for motifs found by that motif finder.

### *S. cerevisiae *ChIP-chip data

Sequence sets used as validation data sets for motif finding were obtained from the *S. cerevisiae *ChIP-chip data from Lee *etal. *[11]. We used a p-value threshold of 0.001 in selecting yeast intergenic regions bound by each of these 10 TFs. This stringent cutoff was the same value used by Lee *et al. *[11] to reduce false positives. Sets of probes that were within the p-value threshold for the 10 selected TFs were sorted into groups. The probes in each group were mapped to unique ORFs, and duplicate ORFs were removed. ORF Sequences were obtained using the annotation and chromosomal sequence from the *Saccharomyces *Genome Database [65,66]. Sequence 1000 bp upstream of translational start was extracted for each ORF. Upstream regions were truncated where there was overlap with the coding regions of neighboring ORFs.

In order to determine the statistical significance of the motifs found by MultiFinder, 10 size-matched randomly selected sequence sets were generated for each of these 10 yeast TFs and submitted to MultiFinder. For example, the set of input sequences for Reb1 contained 80 upstream regions ranging in size from 108 to 1000 bp. Each random set for Reb1 also contained 80 upstream regions and each one of these regions was the same length as its counterpart in the Reb1 test set. Thus, each random sequence set contained the same number of sequences with same total number of base pairs and each sequence within the random set was size-matched to one of the sequences in the Reb1 motif finding sequence set. The geometric mean and range of the group specificity scores of all the motifs found in all the corresponding size-matched random sequence sets were calculated. Similarity between the previously identified motif and the motif identified by MultiFinder was calculated with CompareACE [21], which uses only the six most informative positions of the first PWM in calculating the Pearson correlation coefficient between two PWMs. A Pearson correlation coefficient of at least 0.7 was used to assign a motif found with MultiFinder to a given TRANSFAC identifier. In cases where a MultiFinder motif matched more than one TRANSFAC motif, the TRANSFAC motif with the highest correlation coefficient with the MultiFinder motif was used to assign the TRANSFAC identifier.

### Clustering of human and mouse tissue-specific oligonucleotide array expression data

Probes for orthologous human and mouse genes that displayed similar expression patterns in the GNF SymAtlas [32] were determined by calculating the Pearson correlation coefficient between the expression profiles across the 34 tissue types that were profiled for both human and mouse. The data set contained 1,681 probe pairs with expression correlation coefficients of at least 0.6. The expression data from the 1,681 human genes were then processed with Cluster 3.0 [67,68]. The data were log-transformed, and then five rounds of median-centering and normalization were performed on both probes and tissues. The normalized expression data were then clustered hierarchically using centroid linkage and the centered correlation similarity metric both by probe and by tissue type. The clustering step generated 18 expression clusters of probes with cluster correlation coefficients of at least 0.6 (see Figure 5). Listed in Additional Data File 1 are lists of the nonredundant RefSeq accession IDs to which we could map the Affymetrix probe IDs for each of these 18 expression clusters, as well as for the skeletal muscle expression cluster resulting from clustering all the human expression data regardless of the expression of the orthologous mouse genes. The lists of RefSeq accession numbers were submitted to SequenceExtractor.pl, which resulted in an average of 204 bp input sequence per gene with a range of ~20–1000 bp over all of the gene expression clusters, and then these sequences were submitted to MultiFinder.pl.

### Extraction of and Determination of Matches to TRANSFAC Motifs

Known TFBS motifs were downloaded from TRANSFAC Professional 7.4 (BIOBASE Biological Databases, Germany), and used for the identification of known motifs among the motifs discovered by MultiFinder. Multiple TFBS matrices were present in TRANSFAC for many of the TFs. Because TRANSFAC appends matrix names with a numerical code that indicates their assessment of the quality of the binding site data, for those TFs for which there were multiple TRANSFAC motifs available, we were able to extracted for each TF its highest quality matrix. Briefly, higher quality motifs are indicated with lower numbers; for example, a matrix denoted "Q1" is of the highest quality category and was generated from experimentally verified binding sites using a standard method for finding binding sites, while a matrix denoted "Q6" motif indicates "no quality assigned", which we conservatively set to be the lowest quality category [69]. In instances where a consensus motif was present for a TF but no matrix with a quality code existed, the consensus motif was used. Only motifs with a "V" designator in the name, indicating that they were generated from vertebrate TFBSs, were selected.

Of the 695 total motifs in TRANSFAC Professional 7.4, we extracted 368 nonredundant vertebrate motifs, including consensus motifs, based on their TRANSFAC quality codes. We used CompareACE [21] to determine if any motifs discovered by MultiFinder matched any of the 368 known TFBS motifs. To permit these motif comparisons, we converted all of the TRANSFAC motifs from position frequency matrices into AlignACE-formatted motif files by generating sets of sequences that closely approximated the original TRANSFAC position frequency matrices. The best match with a correlation coefficient of at least 0.6 was used to name the discovered motif. We used the more permissive 0.6 correlation coefficient threshold for the motifs found in these gene expression clusters as compared to the more stringent 0.7 correlation coefficient threshold for the motifs found in the yeast ChIP-chip data sets, since we expected that the mammalian gene expression data sets would tend to be less highly enriched for a given TFBS motif than the yeast ChIP-chip data sets. Some motifs found by the motif finders had multiple matches better than a 0.6 correlation coefficient; in such a case, the graphical view (.svg file) of the motifs retains the name of only that known motif with the best correlation, while the text output (.stat file) retains the names of all known motifs with a correlation coefficient of at least 0.6. A listing of the TRANSFAC motif names that were used to generate each of the AlignACE-formatted motifs is provided in Additional Data File 2.

Our hypothesis was that sequence orthologs with highly correlated expression between human and mouse were likely to be functional orthologs. Thus, in order to identify the TFs in the GNF expression data that were functional orthologs, we selected those probes that showed highly correlated (0.6 correlation coefficient) gene expression between human and mouse. The human GNF probes for genes for which mouse ortholog probes were known (11,186 human probes) were submitted to the Affymetrix NetAffx website in order to identify the probes for genes with known TF activity. The TRANSFAC Professional 8.0 database was then queried for matrices that correspond to the DNA binding site motifs for genes with known TF activity in the GNF expression data. This resulted in 29 TFBS matrices, 27 of which were present in the list of motifs extracted from TRANSFAC Professional 7.4 and thus were considered in motif comparisons.

## Implementation

MultiFinder is a command line program written in Perl using the ActiveState 5.8.0 distribution. The XML::Writer and IO::File modules were used in addition to the standard Perl modules. MultiFinder consists of six source modules and two scripts. The two scripts are SequenceExtractor.pl and MultiFinder.pl. These scripts extract sequence and find motifs respectively. On an Athlon MP 2000+ CPU running at 1.6 GHz with 2 Gb RAM, SequenceExtractor takes nearly 5 minutes to extract the conserved regions and background files and to generate a fifth-order Markov model for the 1000 bp upstream regions of 30 genes belonging to the test set and 8000 genes belonging to the background. MultiFinder takes 23:57 hours to perform a search of 36 sequences with 6870 total base pairs of sequence using all four motif finders at all widths between 6 and 18 bp.

## List of abbreviations used

TF, transcription factor; BS, binding site; TFBS, transcription factor binding site; ChIP, chromatin immunoprecipitation; PWM, position weight matrix.

## Authors' contributions

BRH downloaded all sequence files, performed all programming and analyses of the raw data, and drafted parts of the manuscript. MLB conceived of the study, participated in the design and analysis, and drafted the manuscript. Both authors read and approved the final manuscript.

## Supplementary Material

Additional file 1 The following additional files are available with the online version of this paper, and also on the Bulyk lab website : a PDF file that shows the performance of MultiFinder with yeast ChIP-chip input set (Additional Figure 1); a PDF file that provides examples of motifs found by MultiFinder that failed the block filtering criteria (Additional Figure 2); a PDF file that provides heat maps of each of the 18 selected gene expression clusters that were examined with MultiFinder (Additional Figure 3); a PDF file that provides sample output from SequenceExtractor (Additional Figure 4); a text file in which we provide lists of the nonredundant RefSeq accession IDs to which we could map the Affymetrix probe IDs for each of these 18 expression clusters (Additional Data File 1); a text file in which we list the TRANSFAC TFBS matrix accession number and abbreviated motif name for each of 368 nonredundant vertebrate TFBS motifs (Additional Data File 2); a text file in which we describe the *.ace motif output files, *.svg graphical output files, and *.stat statistical files, available at , for all known and novel motifs discovered by MultiFinder within the examined tissue-specific expression clusters, that passed our block filtering criteria and that had group specificity scores that were more significant than the geometric mean of the matched randoms, along with the geometric means and standard deviations of the group specificity scores for motifs resulting from the five sets size-matched randoms for each expression cluster (Additional Data File 3); a PDF file in which we provide tables supporting our word frequency analysis for each of nine different classes of genomic sequence windows from the human genome (Additional Table 1). In addition, on the Bulyk lab website , we provide via an academic license the MultiFinder, SequenceExtractor, and BlockFilter software and sample input and output files, and a PDF file in which we provide instructions for the installation and usage of the MultiFinder, SequenceExtractor and BlockFilter programs. Click here for file

## Figures and Tables

**Table 1:** Comparison of different regions of sequence conservation in the muscle related genes. **(A) **Number of experimentally verified TFBSs found in the conserved regions of different sizes. The Wasserman conserved regions were taken from Wasserman *et al. *[15]; note that the regions were identified using a Bayesian alignment method between human and mouse, while the upstream regions that we call conserved are from the UCSC Bioinformatics Site [29,30]. The 1, 2, 5 kb conserved regions were generated using the blastz alignment of the human (hg16) and mouse (mm4) taken from the UCSC Bioinformatics Site. The name of each TF family is listed next to the number of known sites taken from Wasserman *et al. *[14]. The number of sites found within the CRMs is listed next. The number of sites found in the sequences generated by SequenceExtractor is listed according to the length of the region. **(B) **Number of sites matching the corresponding TFBS motif, at a Pearson correlation coefficient threshold of 0.6, within each of the regions shown in **(A)**. **(C) **Relative enrichment of each TFBS motif in the specified regions, normalized to the CRMS. Specifically, the enrichment is calculated by dividing the frequency of the motif in the region of interest by its frequency in the CRMs.

**(A) Experimentally verified transcription factor binding sites.**					
**TF**	**Known Sites**	**Wasserman Conserved Sites**	**1 kb Upstream Conserved Sites**	**2 kb Upstream Conserved Sites**	**5 kb Upstream Conserved Sites**

Mef2	21	16	11	11	11
Myf	24	18	14	14	14
Sp1	24	19	16	16	16
SRF	16	12	10	10	10
Tef	9	9	6	6	6

**(B) Sites matching PWM.**					

**TF**		**Wasserman Conserved Sites**	**1 kb Upstream Conserved Sites**	**2 kb Upstream Conserved Sites**	**5 kb Upstream Conserved Sites**

Mef2		25	13	15	17
Myf		78	36	44	91
Sp1		79	43	54	90
SRF		31	22	22	32
Tef		10	7	8	13

**(C) Relative enrichment of PWM matches in sequence windows.**					

**TF**		**Wasserman Conserved Sites**	**1 kb Upstream Conserved Sites**	**2 kb Upstream Conserved Sites**	**5 kb Upstream Conserved Sites**

Mef2		1.00	1.16	1.00	0.57
Myf		1.00	1.03	0.94	0.97
Sp1		1.00	1.21	1.14	0.95
SRF		1.00	1.59	1.19	0.86
Tef		1.00	1.56	1.34	1.08

**Table 2:** Performance of AlignACE, BioProspector, MDscan and MEME on mammalian skeletal muscle CRMs. Results are shown for the five TFs, Mef2, Myf, Sp1, SRF and Tef, with experimentally verified binding sites within these CRMs. A correlation coefficient cutoff of 0.6 was used in the merging of highly similar discovered motifs (see **Methods**). **(A) **Results before block filtering. The number in parentheses next to the rank of the motif indicates the number of times the motif was found in five MultiFinder runs on this CRM sequence set, and the corresponding group specificity scores and ranks are for the highest ranking motif, from the five runs, matching the previously described motifs [35] for these TFs. **(B) **Results following the removal of motifs that failed our block filtering step. The numbers in parentheses next to the column header "Rank" are the range of number of motifs found by that motif finder following motif merging from the five runs. The ranking of the motif based on group specificity and the group specificity score are reported for each motif. In both Tables 2A and 2B, below the results for each of these five TFs, the geometric mean and range of group specificity scores from the five size-matched randomly selected sequence sets (see **Methods**) are listed for each motif finder for comparison.

**(A) Without Block Filtering**								
	**AlignACE**		**BioProspector**		**MDscan**		**MEME**	
	Rank	Group	Rank	Group	Rank	Group	Rank	Group

**Mef2**	1(4)	2.40 × 10^-16^	-	-	1(5)	5.00 × 10^-8^	4(5)	6.90 × 10^-12^
**Myf**	3(5)	4.10 × 10^-8^	-	^-^	3(5)	5.40 × 10^-2^	24(5)	1.40 × 10^-4^
**Sp1**	4(2)	3.80 × 10^-8^	1(4)	1.10 × 10^-8^	2(5)	1.70 × 10^-3^	6(5)	3.60 × 10^-11^
**SRF**	3(4)	4.10 × 10^-8^	-	-	-	-	29(5)	7.20 × 10^-4^
**Tef**	-	-	-	-	-	-	63(5)	2.20 × 10^-2^

**Mean**	4.52 × 10^-3^		2.40 × 10^-7^		8.59 × 10^-5^		1.02 × 10^-3^	
**Range**	6.90 × 10^-1^	7.20 × 10^-8^	1.40 × 10^-1^	2.50 × 10^-24^	2.80 × 10^-1^	1.40 × 10^-9^	3.20 × 10^-1^	1.00 × 10^-29^

**(B) After Block Filtering**								

	**AlignACE**		**BioProspector**		**MDscan**		**MEME**	

	Rank (26–38)	Group	Rank (1)	Group	Rank (4)	Group	Rank (112)	Group

**Mef2**	1	2.40 × 10^-16^	-	-	1	5.00 × 10^-8^	4	6.90 × 10^-12^
**Myf**	3	4.10 × 10^-8^	-	^-^	3	5.40 × 10^-2^	24	1.40 × 10^-4^
**Sp1**	4	3.80 × 10^-8^	1	1.10 × 10^-8^	2	1.70 × 10^-3^	6	3.60 × 10^-11^
**SRF**	2	4.10 × 10^-8^	-	-	-	-	29	7.20 × 10^-4^
**Tef**	-	-	-	-	-	-	49	2.20 × 10^-2^

**Mean**	3.32 × 10^-3^		6.48 × 10^-8^		8.59 × 10^-5^		9.30 × 10^-4^	
**Range**	6.90 × 10^-1^	6.50 × 10^-7^	1.00 × 10^-1^	2.50 × 10^-24^	2.80 × 10^-1^	1.40 × 10^-9^	3.70 × 10^-1^	1.00 × 10^-29^
