# Supplementary material for: Meta-analysis discovery of tissue-specific DNA sequence motifs from mammalian gene expression data
Source: BMC Bioinformatics. 2006 Apr 27;7:229. doi: 10.1186/1471-2105-7-229 (PMC1522027; doi:10.1186/1471-2105-7-229)
Supplement: Additional file 1 — The following additional files are available with the online version of this paper, and also on the Bulyk lab website : a PDF file that shows the performance of MultiFinder with yeast ChIP-chip input set (Additional Figure 1); a PDF file that provides examples of motifs found by MultiFinder that failed the block filtering criteria (Additional Figure 2); a PDF file that provides heat maps of each of the 18 selected gene expression clusters that were examined with MultiFinder (Additional Figure 3); a PDF file that provides sample output from SequenceExtractor (Additional Figure 4); a text file in which we provide lists of the nonredundant RefSeq accession IDs to which we could map the Affymetrix probe IDs for each of these 18 expression clusters (Additional Data File 1); a text file in which we list the TRANSFAC TFBS matrix accession number and abbreviated motif name for each of 368 nonredundant vertebrate TFBS motifs (Additional Data File 2); a text file in which we describe the *.ace motif output files, *.svg graphical output files, and *.stat statistical files, available at , for all known and novel motifs discovered by MultiFinder within the examined tissue-specific expression clusters, that passed our block filtering criteria and that had group specificity scores that were more significant than the geometric mean of the matched randoms, along with the geometric means and standard deviations of the group specificity scores for motifs resulting from the five sets size-matched randoms for each expression cluster (Additional Data File 3); a PDF file in which we provide tables supporting our word frequency analysis for each of nine different classes of genomic sequence windows from the human genome (Additional Table 1). In addition, on the Bulyk lab website , we provide via an academic license the MultiFinder, SequenceExtractor, and BlockFilter software and sample input and output files, and a PDF file in which we provide instructions for the installation and usage of [file 1471-2105-7-229-S1.pdf]

Regulator	Logo of Identified Motif	TRANSFAC Logo	Enrichment	Group Specificity Scores	
Ungapped Binding Site			ChIP-chip	Test Set	Matched Randoms
Gcn4	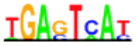	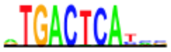	5.00×10^{-39}	2.10×10^{-38}	7.91×10^{-4} (7.50×10^{-1} - 6.00×10^{-22})
Pho4	Not Found	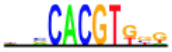	1.00×10^{-1}	1.00×10^{-20}	4.92×10^{-4} (8.50×10^{-1} - 6.90×10^{-21})
Reb1	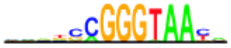	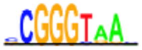	6.00×10^{-54}	3.30×10^{-70}	1.04×10^{-3} (6.40×10^{-1} - 2.00×10^{-20})
Swi4	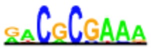	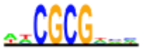	2.00×10^{-9}	1.90×10^{-29}	4.88×10^{-4} (7.40×10^{-1} - 5.10×10^{-22})
Yap1	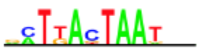	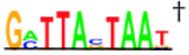 †	9.00×10^{-11}	2.70×10^{-22}	2.17×10^{-2} (8.80×10^{-1} - 9.70×10^{-6})
Gapped Binding Site					
Abf1	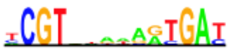	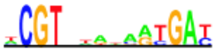	8.00×10^{-101}	2.50×10^{-124}	6.04×10^{-4} (5.80×10^{-1} - 5.30×10^{-43})
Gal4	Not Found	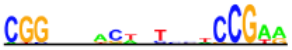	1.00×10^{-3}	2.20×10^{-15}	7.77×10^{-4} (6.30×10^{-1} - 2.00×10^{-19})
HSF1	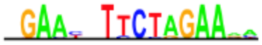	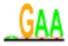	6.00×10^{-21}	6.70×10^{-32}	2.63×10^{-4} (7.30×10^{-1} - 4.90×10^{-23})
Mcm1	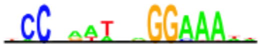	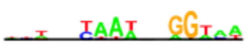	3.00×10^{-18}	1.20×10^{-51}	3.05×10^{-4} (8.90×10^{-1} - 9.20×10^{-20})
Rap1	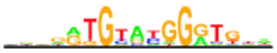	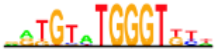	3.00×10^{-24}	7.70×10^{-84}	1.99×10^{-4} (7.10×10^{-1} - 1.00×10^{-21})

Additional Figure 1

Performance of MultiFinder with yeast ChIP-chip input sets. The logo for the highest scoring motif for each TF ("regulator") is shown next to the corresponding TRANSFAC [1,2] logo. The motif enrichment scores as determined by Lee *et al.* [3] are listed next to the corresponding motif. In order to determine the statistical significance of the motifs found by MultiFinder, 10 size-matched randomly selected sequence sets were generated for each of the 10 TFs and submitted to MultiFinder (see **Materials and methods**). The group specificity score for the best scoring motif in the test set is shown next to the geometric mean and range of scores for all of the motifs from 10 randomly generated size-matched sequence sets. † The Yap1 motif was not found in TRANSFAC; instead, the depicted motif was obtained from Cohen *et al.* [4]. Note that HSF1 binds as a trimer and that the TRANSFAC HSF motif corresponds to the binding site for just one monomer.

References for Additional Figure 1:

1. Matys V, Fricke E, Geffers R, Gossling E, Haubrock M, Hehl R, Hornischer K, Karas D, Kel A, Kel-Margoulis O, et al.: **TRANSFAC: transcriptional regulation, from patterns to profiles**. *Nucleic Acids Res.* 2003, **31**:374-378.
2. http://www.cognia.com.
3. Lee T, Rinaldi N, Robert R, Odom D, Bar-Joseph Z, Gerber G, Hannett N, Harbison C, Thompson C, Simon I, et al.: **Transcriptional regulatory networks in *Saccharomyces cerevisiae***. *Science* 2002, **298**:799-804.
4. Cohen BA, Pilpel Y, Mitra RD, Church GM: **Discrimination between Paralogs using Microarray Analysis: Application to the Yap1p and Yap2p Transcriptional Networks**. *Mol. Biol. Cell* 2002, **13**:1608-1614.
